# Prenatal Programming of Human Neurological Function

**DOI:** 10.1155/2011/837596

**Published:** 2011-04-05

**Authors:** Curt A. Sandman, Elysia P. Davis, Claudia Buss, Laura M. Glynn

**Affiliations:** ^1^Women and Children's Health and Well-Being Project, Department of Psychiatry and Human Behavior, University of California, Orange, CA 92868, USA; ^2^Department of Pediatrics, University of California, Irvine, Orange, CA 92868, USA; ^3^Crean School of Health and Life Sciences, Chapman University, Orange, CA 92866, USA

## Abstract

The human placenta expresses the genes for proopiomelanocortin and the major stress hormone, corticotropin-releasing hormone (CRH), profoundly altering the “fight or flight” stress system in mother and fetus. As pregnancy progresses, the levels of these stress hormones, including maternal cortisol, increase dramatically. These endocrine changes are important for fetal maturation, but if the levels are altered (e.g., in response to stress), they influence (program) the fetal nervous system with long-term consequences. The evidence indicates that fetal exposure to elevated levels of stress hormones (i) delays fetal nervous system maturation, (ii) restricts the neuromuscular development and alters the stress response of the neonate, (iii) impairs mental development and increases fearful behavior in the infant, and (iv) may result in diminished gray matter volume in children. The studies reviewed indicate that fetal exposure to stress peptides and hormones exerts profound programming influences on the nervous system and may increase the risk for emotional and cognitive impairment.

## 1. Introduction

The Developmental Origins of Disease or Fetal Programming model predicts that early exposures to threat or adverse conditions have lifelong consequences that result in poor health outcomes [1]. The vast majority of the studies in support of the programming model in human beings are retrospective and most relied on surrogate measures of early experience such as low birth weight or preterm birth. The retrospective studies and the growing number of prospective studies have reported that fetuses exposed to maternal stress at various times during gestation are at greater subsequent risk for cardiovascular and metabolic disorders that shorten lifespan. In addition, the studies reviewed here indicate that fetal exposure to peptides and hormones from the maternal HPA and placental stress system exerts profound programming influences on the brain. 

Programming is a process by which a stimulus or exposure during a critical developmental period has a long-lasting or permanent influence on the brain, behavior, and risk for disease. Critical periods are defined by epochs of rapid cell division within an organ and different organs develop at rates and at different times [2]. During these periods of rapid cell division, fetal organs are especially vulnerable to perturbations such as stress [3]. Because tissues develop in a specific sequence, the timing of exposures determines the nature of the programmed effect [1].

## 2. The Hypothalamic Pituitary Adrenal (HPA) and Placental Stress System

**Figure 1:**
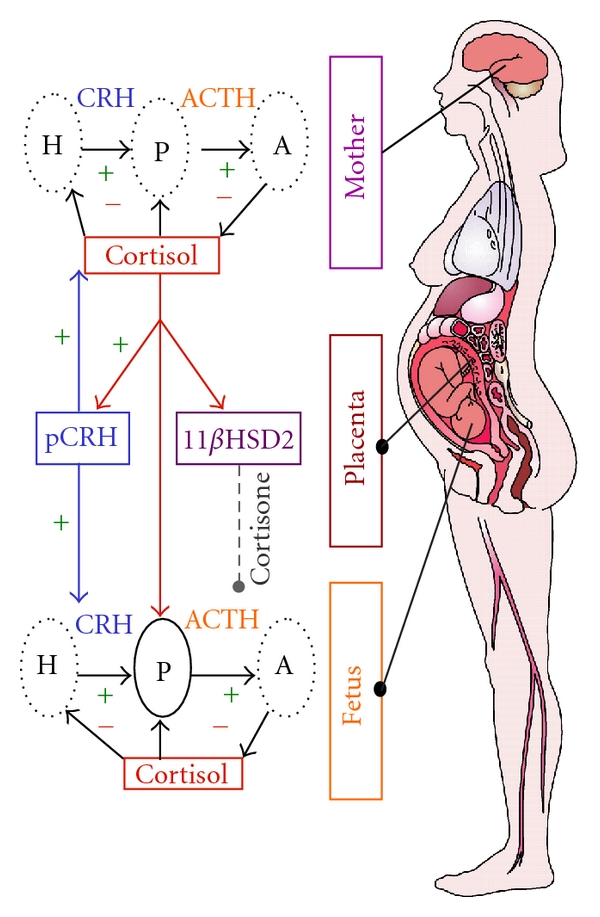
The regulation of the HPA axis changes dramatically over the course of gestation with profound implications for the mother and the fetus. One of the most significant changes during pregnancy is the development of the placenta, a fetal organ with significant endocrine properties. During pregnancy, CRH is released from the placenta into both the maternal and fetal compartments. In contrast to the negative feedback regulation of hypothalamic CRH, cortisol *increases* the production of CRH from the placenta. Placental CRH (pCRH) concentrations rise exponentially over the course of gestation. In addition to its effects on pCRH, maternal cortisol passes through the placenta. However, the effects of maternal cortisol on the fetus are modulated by the presence of p11*β*HSD2 which oxidizes it into an inactive form, cortisone. Activity of this enzyme increases as pregnancy advances and then drops precipitously so that maternal cortisol is available to promote maturation of the fetal lungs, central nervous system, as well as other organ systems.

One system profoundly influenced during human pregnancy is the “fight or flight" stress system because of the growth and development of the placenta [4] (Figure 1). The placenta expresses the genes for the major stress hormones, CRH (hCRHmRNA) and proopiomelanocortin, the precursor for ACTH and beta-endorphin (BE). All of these stress hormones increase as pregnancy advances, but the exponential increase in placental CRH (pCRH) in maternal plasma is especially dramatic, reaching levels observed only in the hypothalamic portal system during physiological stress [5]. Moreover, in contrast to the well-known negative feedback regulation of hypothalamic CRH, cortisol stimulates the expression of hCRHmRNA in the placenta, establishing a positive feedback loop that allows for the simultaneous increase of pCRH, ACTH, BE, and cortisol over the course of gestation. The difference in behavior of the CRH gene in the placenta and hypothalamus is due to the expression of different transcription factors, coactivators, and corepressors in these two tissues [6]. The increase of pCRH especially over the latter part of human gestation plays a fundamental role in the organization of the fetal nervous system [7] and in maternal adaptation during pregnancy, including influencing the timing of the onset of spontaneous labor and delivery [8, 9]. 

The effects of stress/HPA and placental axis hormones are modulated by the activities of binding proteins and enzymes. For example, concurrent with increases in pCRH, maternal CRH-binding protein rises and then falls abruptly near the end of gestation [8]. Maternal plasma cortisol-binding globulin (CBG) levels also change across pregnancy. CBG is stimulated by estrogen, and these levels increase progressively with advancing gestation until the end of gestation when there is a significant decline in CBG [10]. The levels of placental 11*β*-HSD2 (which oxidizes cortisol into its inactive form, cortisone) [11] rise as gestation progresses before falling precipitously near term ensuring maturation of the fetal lungs, CNS, and other organ systems in full term births [12, 13].

 The maternal-fetal endocrine changes are adaptive and important for fetal maturation but if the levels are elevated, for instance in response to stress, it can affect the trajectory of fetal development. Compelling evidence from the Western Spadefoot toad implicates the HPA system and particularly CRH, in the control of the rate of development [14–17]. Rapidly evaporating pools of desert water result in elevation of CRH in the median eminence of the tadpole precipitating metamorphic climax to escape imminent peril. If the CRH response is blocked during environmental desiccation, then the rate of development is arrested and the tadpole's survival is compromised. This remarkable surveillance and response system has evolved and is conserved so that many species including the human fetus can detect threats to survival and adjust its developmental trajectory [8, 18–20]. The placenta collects information from its maternal host to prepare the fetus for postnatal survival [21]. If the fetal/placental unit detects stress signals from the maternal environment (e.g., cortisol), the “placental clock" [8] may be advanced by activation of the promoter region of the CRH gene which initiates the placental synthesis of the “master” stress hormone, pCRH [22]. The rapid increase in circulating pCRH initiates the cascade of events resulting in myometrial activation that increases the risk of preterm birth. In parallel, the fetus adjusts its developmental trajectory and/or modifies its nervous system to ensure survival in a potentially hostile environment. Survival under these circumstances, however, is associated with compromised motor, cognitive, and emotional function [23, 24] and reduced region-specific brain gray matter volume [25–27].

It is important to acknowledge that there are vast differences in reproductive physiology as well as in the trajectory of fetal brain development, even in very closely related species, such as humans and nonhuman primates. These differences limit the validity of generalizing to humans from animal models [28]. For instance, placental CRHmRNA is found only among some primates, and, even among nonhuman primates, the timing of synthesis and release of pCRH is different than for humans (and great apes).

## 3. Programming the Human Nervous System

The human fetal brain is a primary target for programming influences because it is undergoing dramatic growth over a prolonged period of time. Between gestational age (GA) 8 and 16 weeks, migrating neurons form the subplate zone, awaiting connections from afferent neurons originating in the thalamus, basal forebrain, and brainstem. Concurrently, cells accumulating in the outer cerebral wall form the cortical plate which eventually will become the cerebral cortex. By gestational week 20, axons form synapses with the cortical plate. This process continues so that, by gestational week 24, cortical circuits are organized [29, 30]. The enormous growth of the nervous system is characterized by the proliferation of neurons. By gestational week 28, the number of neurons in the human fetal brain is 40% greater than in the adult [30–33]. The rate of synaptogenesis reaches an astonishing peak so that at gestational week 34 through 24 months postpartum, there is an increase of 40,000 synapses per second [34]. Thus, prenatal life is a time of enormous neurological change and the nervous system is particularly vulnerable both to organizing and disorganizing programming influences. 

Recently, prospective studies in humans have documented the developmental consequences for the nervous system of exposures to stressful intrauterine conditions [35–51]. These studies clearly have shown that fetal exposure to elevated gestational stress and stress hormones has significant and largely negative consequences for fetal, infant, and child neurological development. Fetal exposure to high levels of stress and stress hormones, especially early in gestation, results in delayed fetal maturation and impaired cognitive performance during infancy and results in decreased brain volume in areas associated with learning and memory in children. The accumulating evidence supports the conclusion that fetal exposure to stress profoundly influences the nervous system with consequences that persist into childhood and perhaps beyond.

## 4. Prenatal Exposure to Stress Influences Human Fetal Behavior

Observation of human fetal behavior provoked by stimulation provides a noninvasive method of assessing brain functioning [52]. Measures of human fetal responding are accepted indicators of fetal maturity [53, 54] that reflect the development and integrity of neural pathways through the cerebral cortex, midbrain, brainstem, vagus nerve, and the cardiac conduction system [55]. We have reported that fetuses of women with elevated pCRH concentrations during the third trimester were less responsive to the presence of a novel stimulus [7] and that fetal heart rate (FHR) habituation was delayed when fetuses were exposed to overexpression of maternal endogenous opiates [56]. These studies illustrate that maternal and placental stress hormones exert acute influences on fetal behavior. Recent studies from our group demonstrate that stress hormone exposures early in gestation exert programming influences on the developmental trajectory of the fetal nervous system. 

In a large cohort of 191 mother/fetal dyads serially evaluated throughout pregnancy, we described the maturational trajectory of the FHR response pattern to a startling stimulus [57]. In this study, we observed that at ~25 weeks of gestation only a small percentage of subjects manifested an observable response but, by ~30 weeks, nearly all subjects showed evidence of stimulus detection. The individual differences at the ~25-week period provided an ideal opportunity to examine programming influences on the fetal nervous system. We found that low pCRH at 15 gestational weeks, but not later, predicted a more mature fetal heart rate pattern at 25 gestational weeks suggesting that the pattern of development was altered by these early exposures [58]. This is evidence that gestational stress exerts programming influences on the developing nervous system that is independent of postnatal experiences.

## 5. Prenatal Stress Influence Neonatal Neurological Status and Stress Regulation

Assessment of the newborn offers an important opportunity to identify the consequences of fetal programming independent of postpartum influences on development. In a study from our group [35], the New Ballard Maturation Score was used to assess physical and neuromuscular maturation of 158 newborns within 24 hours after birth. This examination of infant maturation is composed of multiple assessments of newborn characteristics that correspond to a metric of developmental age. Specifically, the neuromuscular and physical characteristics of the newborn are rated and consist of measures of muscle tone, distinct posture, and angles of resistance in key muscle groups. The results of this study provided evidence that fetal exposure to increases in levels of maternal cortisol at 15 and at 19 weeks of gestation and increases in levels of pCRH at 31 weeks' gestation were associated with significant decreases in newborn physical and neuromuscular maturation. These effects were observed after controlling for length of gestation, indicating that fetal exposure to stress hormones programs neonatal neuromuscular maturation independent of gestational age. 

Decreased scores on newborn neuromuscular maturity have been associated with abnormalities detected with MRI in newborns, including basal ganglia and white matter lesions, as well as motor abnormalities that persist until age 4 in childhood [59, 60]. Moreover, in newborns regarded as healthy by obstetric and pediatric staff, deviant patterns on neurological examinations (including measures of posture, movement, tone, reflexes, and some behavior) have been associated with newborn cranial ultrasound abnormalities, including thalamic and periventricular densities and intraventricular hemorrhaging [61]. Our report [35] indicates that fetal exposure to elevated levels of maternal stress hormones early in pregnancy and placental stress hormones late in pregnancy was associated with neonatal measures of maturation that reflect neurological development.

Prenatal exposure to maternal stress hormones similarly programs the development of the fetal HPA axis with consequences for neonatal functioning. Recently we reported [36] in a sample of 116 mothers and their healthy full term infants, assessed at five gestational intervals and at 24 hours after birth, that prenatal maternal cortisol and psychosocial stress each exerted influences on neonatal stress regulation and these influences were dependent upon the gestational period during which the fetus was exposed. Specifically elevated maternal cortisol early in gestation was associated with slower neonatal behavioral recovery from the painful stress of a heel-stick procedure. Elevated maternal cortisol during the second half of gestation was associated with a larger and more prolonged neonatal cortisol response to stress. The data from this study are consistent with evidence that prenatal exposure to synthetic glucocorticoids during the late second and early third trimester is associated with an amplified cortisol response to stress among healthy full term neonates [62]. Together, these data provide evidence that gestational exposure to excess glucocorticoids alters the developmental trajectory of the fetal HPA axis with consequences for postnatal stress regulation. Alterations to neurological systems at different times during fetal development resulting from prenatal exposures may determine the neonate's ability to respond behaviorally and physiologically to stressors in the postnatal environment. It is plausible that neonates who are more reactive may carry a greater risk for the development of behavioral inhibition and anxiety during infancy and childhood.

## 6. Prenatal Stress Influences Infant and Toddler Behavior

There is growing acceptance that prenatal exposure to various stressors, including experiential assessment of stress and anxiety, is associated with behavioral and emotional disturbances during infancy and childhood that are independent of birth outcome and postpartum maternal stress or depression [37, 40–46, 51, 63]. For instance, prenatal exposure to elevated levels of maternal psychosocial stress and stress hormones is associated with behavioral and emotional disturbances during infancy and childhood that are independent of birth outcome and postpartum maternal stress or depression [40, 41, 45, 46]. Our studies have shown that elevated levels of prenatal maternal anxiety and depression were associated with increased infant fearful temperament after controlling for the influence of postpartum maternal state in both maternal report and laboratory observational measures of temperament [42, 43]. These studies are consistent with the possibility that prenatal exposure to elevated levels of maternal stress signals contributes to the development of a more fearful temperament and thus increases vulnerability to the development of anxiety [37, 42].

Few studies have examined the effects of prenatal stress on cognitive development. Although there is evidence that maternal self-report of elevated stress, depression, and anxiety during the prenatal period is associated with delayed infant cognitive and neuromotor development [42, 47] and that these deficits may persist into adolescence [48]; the findings across studies are not consistent [50, 54]. In the largest study conducted (125 subjects) with repeated evaluations at five prenatal intervals and three intervals during infancy, we reported that the consequences of fetal exposure to elevated maternal cortisol and pregnancy-specific anxiety (PSA) were dependent upon when during gestation exposure to these two indicators of stress was elevated [38]. Fetal exposure to higher levels of cortisol early in pregnancy resulted in significantly lower scores on measures of mental development. Conversely, elevated maternal cortisol late in gestation was associated with significantly higher scores on measures of mental development. Similar results were observed for levels of maternal PSA. Despite the similar effects of maternal cortisol and anxiety on infant cognition at one year of age, these two measures of prenatal stress were not related and exerted independent effects on developmental outcomes. 

The findings linking cortisol to infant cognitive development are consistent with its function in the maturation of the human fetus. As described above, early in pregnancy the fetus is partially protected from maternal cortisol because it is oxidized and inactivated by placental 11*β*-HSD2. However, because placental 11*β*-HSD2 is only a partial barrier, excessive synthesis and release of maternal cortisol exposes the fetus to concentrations that may have detrimental neurological consequences. We have found that elevated maternal cortisol early in gestation is associated with delayed neonatal and infant maturation. As pregnancy advances toward term, fetal exposure to elevated cortisol is necessary for maturation of the fetal nervous system and lungs [64]. Fetal exposure to cortisol during the third trimester is facilitated by the sharp drop in placental 11*β*-HSD2 which allows a greater proportion of maternal cortisol to cross the placental barrier [49, 65]. Our findings indicate that fetal exposure to maternal cortisol late in gestation has adaptive consequences for the developing nervous system and this is reflected in increased mental proficiency in the infant.

## 7. Prenatal Stress Influences Brain Morphology

Retrospective studies have suggested that the quality of the prenatal environment influences the trajectory of brain development [25, 66]. In the first prospective study to evaluate the consequences of prenatal maternal anxiety on child brain development, we reported that 6–9-year-old children of women who reported high levels of PSA early in gestation had region-specific reductions in gray matter volume [39]. Structural MRI indicated that high levels of maternal PSA during the early second trimester of pregnancy was associated with volume reductions in the prefrontal cortex, the premotor cortex, the medial temporal lobe, the lateral temporal cortex, and the postcentral gyrus as well as the cerebellum extending to the middle occipital gyrus and the fusiform gyrus. These associations were independent of postpartum measures including maternal stress. The affected brain regions are known to sustain a range of cognitive functions. Specifically, the prefrontal cortex is involved in executive cognitive functions such as reasoning, planning, attention, working memory, and some aspects of language [67]. Structures in the medial temporal lobe, including areas connected to the hippocampus (entorhinal, perirhinal, and parahippocampal cortex), constitute a medial temporal lobe memory system [68]. The temporal polar cortex is involved in social and emotional processing including recognition and semantic memory [69, 70]. A network in the temporal-parietal cortex consisting of the middle temporal gyrus, the superior temporal gyrus and the angular gyrus, has been shown to be important in processes related to auditory language processing in children [71]. Brain systems involved in language learning including the inferior frontal gyrus, the middle temporal gyrus, and the parahippocampal gyrus also are reduced in children “exposed" to high levels of PSA [72]. Thus, such altered patterns of brain development may underlie cognitive impairment observed in infants exposed to high levels of PSA [38]. Although these results do not implicate the HPA and placental axis directly, they add strong support to the argument that the nervous system is a vulnerable target for programming influences and may increase the risk of developing emotional and cognitive disorders.

## 8. Conclusions

**Figure 2:**
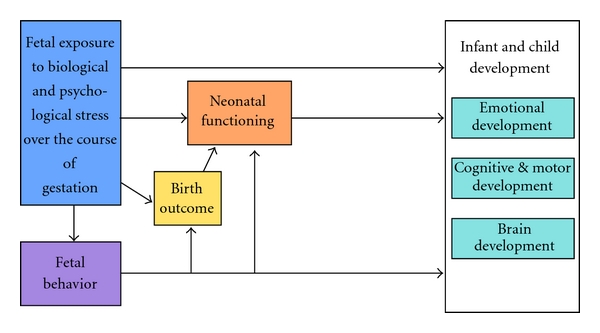
Schematic representation of the psychobiological stress, fetal programming model that guides our research program. Fetal exposure to stress can influence infant/child development directly or indirectly (through fetal behavior, birth outcomes, and neonatal functioning).

As illustrated in Figure 2, fetal exposure to maternal biological and psychosocial stress can produce a complex, interrelated series of consequences throughout early development that may persist throughout the lifetime. The studies reviewed indicate that maternal responses to adversity influence fetal behavior, birth outcome, and neonatal and child outcomes. The model indicates that prenatal maternal adversity alters the fetal developmental trajectory and regulates birth outcome. There are direct programming effects of maternal adversity on developmental outcomes and indirect effects or effects that are mediated by birth outcome. Direct effects may be observed as early as the neonatal period. However, some of the consequences of prenatal exposure to adversity will not be detected until later in life. For example, prenatal maternal stress may alter the trajectory of fetal brain development resulting in developmental impairments that only emerge as that capacity develops. Thus, for example, as cognitive functions come “on line” we may begin to detect delays that were not apparent previously. In other cases, the effects of exposure to maternal stress signals could operate through an indirect route. For instance, exposure to prenatal adversity can disrupt the developmental trajectory of the fetus. The consequences of disruptions to the fetal developmental trajectory may increase the vulnerability to stress later in life. Through this indirect route, the influence of prenatal stress is magnified by the limited abilities to cope with later adversity. 

The precise mechanism by which the pregnant woman communicates her psychological state of stress or adversity to her fetus is unknown. The relation between psychosocial measures of stress and the HPA axis during pregnancy is low and nonsignificant [38, 73, 74] suggesting that fetal exposure to stress hormones alone is not the mechanism of communication. Findings from animal studies indicate that poor maternal care in very early development alters the methylation status of the NGFI-A binding site in a region of the Nr3c1 promoter responsible for control of hippocampal glucocorticoid receptor expression supporting an epigenetic effect with direct implications for areas of the nervous system involved with mental development [75]. These findings and others reporting persisting altered BDNF expression in the prefrontal cortex related to early adversity [76] provide possible routes of maternal influence on fetal and early infant neurological development with profound implications for infant/child adjustments to life challenges.

There are plausible routes of maternal biological stress influencing the fetal nervous system. Concentrations of pCRH in maternal circulation reflect the integration of numerous stress signals (e.g., nutritional deprivation, immune markers, hypertension, etc.), not just those related to psychological stress [77–79]. As such, fetal exposure to pCRH may be a final common pathway for the “programming” effects of adversity on the developing nervous system. Elevated concentrations of pCRH may affect directly the developing brain by upregulation (e.g., amygdala) or downregulation (e.g., hippocampus) of CRH receptors in the brain [80]. Exogenously administered CRH has been shown to increase limbic neuronal excitation leading to seizures [80–82] and may participate in mechanisms of neuronal injury [81, 83]. CRH has neurotoxic effects on hippocampal neurons [81, 84–87], and these effects seem to be more pronounced in the immature hippocampus [81, 86, 88]. 

pCRH may influence the fetal nervous system indirectly by stimulating production of fetal cortisol. The sources of cortisol in the fetal compartment are from the fetal and maternal adrenal glands. Because the CRH_1_ receptor is present in human fetal adrenal tissue from mid-gestation onwards [89], maternal CRH can stimulate fetal cortisol production. Moreover, although fetal exposure to maternal cortisol is regulated by placental 11*β*-HSD2, this enzyme is only a partial barrier so that a proportion of maternal cortisol passes through the placenta [90, 91]. Glucocorticoid receptors are present throughout the central nervous system [92–95] and glucocorticoids easily pass through the blood-brain barrier [96] and influence multiple brain regions, including the hippocampus, amygdale, and prefrontal cortex. At high concentrations, cortisol may inhibit growth and differentiation of the developing nervous system, and considerable evidence indicates that glucocorticoids are neurotoxic to hippocampal CA3 pyramidal cells [97, 98]. In embryonic hippocampal neurons corticosterone induces neuronal death [99]. Delayed myelination has been reported in the corpus callosum in association with prenatal exogenous glucocorticoid exposure [100], which is consistent with findings that prenatal stress exposure affects the size of the corpus callosum [101].

Maternal exposure and response to adversity exerts profound influences on the fetal nervous system. These effects “program” the nervous system with consequences that persist throughout the lifespan. The precise mechanisms of communication between mother and fetus are not known, but the roles of pCRH and maternal cortisol are becoming apparent and will be the focus of future research.
